# Evaluating Individual Students' Perceptions of Instructional Quality: An Investigation of their Factor Structure, Measurement Invariance, and Relations to Educational Outcomes

**DOI:** 10.3389/fpsyg.2016.00110

**Published:** 2016-02-08

**Authors:** Ronny Scherer, Trude Nilsen, Malte Jansen

**Affiliations:** ^1^Faculty of Educational Sciences, Centre for Educational Measurement at the University of Oslo (CEMO), University of OsloOslo, Norway; ^2^Department of Teacher Education and School Research, Faculty of Educational Sciences, University of OsloOslo, Norway; ^3^German Institute for International Educational ResearchBerlin, Germany

**Keywords:** bifactor models, classroom perceptions, exploratory structural equation modeling, instructional quality, measurement invariance, PISA 2012

## Abstract

Students' perceptions of instructional quality are among the most important criteria for evaluating teaching effectiveness. The present study evaluates different latent variable modeling approaches (confirmatory factor analysis, exploratory structural equation modeling, and bifactor modeling), which are used to describe these individual perceptions with respect to their factor structure, measurement invariance, and the relations to selected educational outcomes (achievement, self-concept, and motivation in mathematics). On the basis of the Programme for International Student Assessment (PISA) 2012 large-scale data sets of Australia, Canada, and the USA (*N* = 26,746 students), we find support for the distinction between three factors of individual students' perceptions and full measurement invariance across countries for all modeling approaches. In this regard, bifactor exploratory structural equation modeling outperformed alternative approaches with respect to model fit. Our findings reveal significant relations to the educational outcomes. This study synthesizes different modeling approaches of individual students' perceptions of instructional quality and provides insights into the nature of these perceptions from an individual differences perspective. Implications for the measurement and modeling of individually perceived instructional quality are discussed.

## Introduction

Instructional quality is considered to be one of the most important predictors of learning outcomes (Seidel and Shavelson, 2007; Creemers and Kyriakides, 2008; Hattie, 2009). Hence, the construct has received much attention in both national and international large-scale assessments (Klieme, 2013; OECD, 2013a). In these assessments, the construct is most often measured by students' perceptions of how teachers support, cognitively activate, and manage their classrooms (Fauth et al., 2014). Although these individual perceptions provide somewhat valid indicators of instructional quality even at the classroom level (Wagner et al., 2013), a number of methodological challenges are involved in their measurement. First, the conceptual definition of “instructional quality” and the measures need to be aligned. This implies that, for instance, the distinction between the three factors of instructional quality, teacher support, cognitive activation, and classroom management should be reflected in students' perceptions. But current research suggests that this distinction is not perfect and may impose the coexistence between general and specific factors (e.g., Charalambous et al., 2014). As a consequence, traditional measurement models may not fully represent the conceptualization of perceived instructional quality, pointing to the need for flexible modeling approaches. Second, on the basis of appropriate measurement models, specific degrees of measurement invariance across groups need to be met in order to examine the differences and similarities in individual students' perceptions (Millsap, 2011). This need has become a major challenge, particularly in international large-scale assessments such as the Programme for International Student Assessment (PISA), which are aimed at comparing countries and educational systems with respect to how instruction is perceived (e.g., Desa, 2014; Rutkowski and Svetina, 2014). Establishing measurement invariance has therefore become essential for comparing students' perceptions of instructional quality across countries.

Against this background, we are aimed at addressing these two major challenges in evaluating perceived instructional quality by: (a) introducing and studying the performance of different latent variable models that are able to account for flawed links between items and factors by introducing cross-loadings on the one hand, and modeling both general and specific factors on the other hand; (b) evaluating the degree to which measurement invariance is met for these modeling approaches.

We use the Australian, Canadian, and US-American data sets of PISA 2012 and apply four modeling approaches to the scales measuring students' perceptions of instructional quality. These approaches can be differentiated by two criteria: whether the models allow for item cross-loadings or not and whether the models differentiate between specific and general factors or not. Whereas models from the confirmatory factor analysis (CFA) family impose strict item-factor relations without cross-loadings, exploratory structural equation models (ESEM) allow such cross-loadings. Within these two broad families of models, both can be specified with and without a nested general factor, resulting in a total of four models that we test: Traditional CFA, bifactor CFA, ESEM, and bifactor ESEM (see Table 1). To examine whether these modeling approaches may affect not only the psychometric properties of the emerging factors, but also substantive conclusions that researchers may draw, we investigate the relations between perceptions of instructional quality and educational outcomes using these four approaches. Thus, this study provides a synergism between advanced methodology in latent variable modeling and the substantive interest of understanding the nature of students' perceptions of instructional quality.

**Table 1:** **Framework of modeling approaches**.

		**Distinction between a general factor and specific factors**	
		**No**	**Yes**
Estimation of cross-loadings	No	CFA	Bifactor CFA
	Yes	ESEM	Bifactor ESEM

*CFA, Confirmatory factor analysis; ESEM, exploratory structural equation modeling*.

### Conceptualizing students' perceptions of instructional quality

There is a consensus in teaching effectiveness research that instructional quality should be considered a multidimensional rather than a unitary construct with at least three core factors: teacher support, cognitive activation, and classroom management (Creemers and Kyriakides, 2008; Klieme, 2013). *Teacher support* includes teachers' practices such as providing extra help when needed, listening to and respecting students' ideas and questions, and caring about and encouraging students (Klusmann et al., 2008). *Cognitive activation* comprises instructional activities, in which students have to evaluate, integrate, and apply knowledge in the context of problem solving (Lipowsky et al., 2009). *Classroom management* involves elements of discipline, dealing with disruptive student behavior, and time-on task management. This factor may be described as actions taken by teachers to ensure an orderly environment and effective use of time during lessons (Klusmann et al., 2008; Van Tartwijk and Hammerness, 2011).

All of these three factors have been shown to be positively related to students' educational outcomes. For instance, a recent study based on the Trends in International Mathematics and Science Study 2011 demonstrated that a safe and orderly environment positively affected student achievement across a number of countries (Martin et al., 2013). Moreover, Dietrich et al. (2015) identified significant relations between teacher support in classrooms and students' intrinsic motivation. Fauth et al. (2014) supported this finding by showing that teacher support and cognitive activation significantly correlated with students' development of subject-specific interest, whereas classroom management was correlated with student achievement. In this context, Klieme et al. (2009) pointed out that a supportive climate is the strongest predictor of students' motivation and affect. Lazarides and Ittel (2012) extended the existing body of research with a study that revealed that individual students' perceptions of instructional quality were associated with their self-concept and attitudes in mathematics. Generally speaking, there is some evidence that instructional quality is related to students' educational outcomes. This finding has become particularly important for evaluating measurement instruments that are designed to assess students' perceptions of instructional quality factors (e.g., Wagner et al., 2013; Fauth et al., 2014). In fact, we will make use of this knowledge about these relations in comparing the performance of different modeling approaches.

### Measuring individual students' perceptions of instructional quality

In educational large-scale assessments such as PISA, students' perceptions of instructional quality most often serve as proxies for instructional quality because seemingly more “objective” methods such as observer ratings cannot be employed in such studies for practical reasons (OECD, 2013b; Wagner et al., 2013; Fauth et al., 2014). Although these proxies do not provide perfectly reliable measures (Greenwald, 1997; Marsh and Roche, 1997), research suggests that student ratings are significantly related to teacher ratings of their instruction, show the same factor structure, and converge if the relations to student outcomes are studied (Roche and Marsh, 2002; Kunter et al., 2008).

An important question that comes along with the use of student ratings concerns the appropriate level of analysis. Lüdtke et al. (2009) clearly pointed out that the decision on the level of analysis depends on the research question posed. In their argumentation, they distinguish between different types of questions that can either deal with (a) the use of students' perceptions of instruction in order to describe individual differences in these perceptions, (b) the characteristics of the learning environment, or (c) the effects of instructional quality on students' educational outcomes. Clearly, if researchers are interested in the effects of learning environments on educational outcomes, the appropriate level of analysis is the classroom level (Marsh et al., 2012). In such a context, students' aggregated perceptions are of interest in order to model climate or contextual effects (Morin et al., 2014; Scherer and Gustafsson, 2015). On the one hand, these aggregated perceptions are considered to be indicators of the characteristics of the environment (e.g., the degree of cognitive activation in a classroom), as they represent students' shared perceptions; individual differences in these perceptions are therefore considered to be sources of error in the measurement of shared perceptions (Lüdtke et al., 2009). As a consequence, studying the relations among instructional quality and educational outcomes by assuming that instructional quality is a characteristic of the learning environment as posed under (b) and (c) requires a multilevel approach with the classroom level as the main focus (Marsh et al., 2012; Scherer and Gustafsson, 2015).

Drawing on the early works by Murray (1938), Stern (1970) and Lüdtke et al. (2009) distinguish between a consensual (i.e., aggregated) and private (i.e., individual) perspective on students' perceptions. Whereas the former refers to a shared understanding of an environment or group, the latter refers to individuals' perceptions of the environment or group. This distinction is based on the assumption that “behavior results from the interplay of environmental influences (…) and individual needs” (Lüdtke et al., 2009, p. 121). As a consequence, the authors further argue that whenever researchers are interested in inter-individual differences between students' perceptions, the individual level is the appropriate level of analysis. As such, individual students' perceptions of instructional quality provide information on differences between students rather than groups (e.g., classrooms or schools). This is in line with Parker et al. (2003) who showed in their meta-analysis that individual perceptions of the learning or working environment are meaningful for motivation, well-being, and performance. The study conducted by Lazarides and Ittel (2012) also confirmed this argument, studying the relations between students' perceived instructional quality, self-concept, and motivation. This perspective on instructional quality focuses on individual differences in students' perceptions, which have also been studied in the context of “psychological climate” in organizational psychology (Parker et al., 2003; Kuenzi and Schminke, 2009).

In the context of PISA, we note that, given the two-stage random sampling design of students (stage 1) and schools (stage 2), questions concerning the effectiveness of teaching and learning environments cannot directly be addressed, as the classroom level as the appropriate level of analysis is missing (Klieme, 2013). Furthermore, we argue that the school-level information available in PISA may not necessarily solve this issue. Specifically, aggregating students' perceptions to the school level without taking into account the classroom level, neglects differences in instructional quality between teachers and classrooms. This major drawback of adopting a multilevel approach with only the school and student level may therefore lead to biases in the estimation of variances and covariances. Nevertheless, the random sampling of students' within schools independent of the classrooms in PISA 2012 provides valuable data in order to study individual differences in students' perceptions.

In light of this argumentation, the present study focuses on the individual level, because we are interested in inter-individual differences in students' perceptions of instructional quality.

### Modeling individual students' perceptions of instructional quality

Along with the operationalization of instructional quality comes the question of whether the three factors of teacher support, cognitive activation, and classroom management represent strictly distinct constructs. But this is not only a conceptual question concerning the construct; it is also a question of how students' perceptions of instructional quality are assessed. For example, when students are asked to rate on “The teacher helps us to learn from mistakes we have made,” an item designed to measure perceived cognitive activation (OECD, 2013b); their perceptions may also relate to teacher support. In fact, cognitively demanding learning environments often require teachers to support students in order to foster learning (Baumert et al., 2010; Jonassen, 2011). From a measurement perspective, this overlap should not only manifest in high correlations between the two factors of perceived instructional quality, but also in an improvement in goodness-of-fit when models are used that take into account that items could belong to more than one factor. Until now, this overlap between the instructional factors has not yet been addressed in the context of educational large-scale assessments. Instead, models were specified in which every item strictly loads on only one factor (most commonly, confirmatory factor analysis, CFA). Theoretically, however, items pertaining to the perceptions of teaching activities that are both related to cognitive activation and teacher support would be expected to load on both factors. Test development based on CFA models suggests excluding such items because cross-loadings would substantially lower model fits. Whereas excluding such items might be reasonable from a measurement perspective, it would compromise the conceptual breadth of cognitive activation and teacher support from a theoretical perspective. For instance, cognitively demanding learning environments often require teachers' support (e.g., by providing immediate feedback). This interdependence should be accounted for in the measurement of perceived instructional quality.

Besides addressing the conceptual overlap between the factors of instructional quality, researchers also pose the question of the extent to which ratings of the classroom environment represent general or specific perceptions of instruction—that is, is there a “halo effect” on students' ratings of instruction or can they well differentiate between different aspects such as the three factors of instructional quality (e.g., Harlen, 2006; Charalambous et al., 2014)? Hence, the question arises whether students are generally able to distinguish between the different factors of instructional quality in their ratings (specific factors) or they only provide overall ratings of instruction irrespective of these factors (general factor). Previous studies showed the three factors of student-perceived instructional quality to be moderately related (e.g., Wagner et al., 2013; Fauth et al., 2014). Disentangling what is general and specific in these perceptions also provides valuable information on students' response styles, which may relate to the specific educational culture they are based in He and van de Vijver (2013). Specifically, when students evaluate aspects of instruction, they might show positive or negative tendencies in their responses, leading to systematic response bias. As a consequence, measurement models are needed that separate these general tendencies from the specific responses to the different aspects of instructional quality. The most prominent models refer to bifactor models, which assume a general factor and uncorrelated specific factors (Reise, 2012). In this regard, the general factor may not necessarily carry substantive meaning in students' ratings but may handle general response tendencies (He and van de Vijver, 2013). In the current study, we examine how these models perform in an applied substantive context.

In the context of international large-scale studies such as PISA, one major goal is to compare individual students' perceptions across countries in order to make inferences on the extent to which these perceptions are determined by cultural or economic factors (He and van de Vijver, 2013; OECD, 2013a). These comparisons among average ratings across countries require that ratings are comparable. In statistical terms, measurement invariance needs to be established (Millsap, 2011). In some previous research on the comparability of perceived instructional quality, full invariance could not be established, thus limiting researchers to reporting outcomes for each country separately or providing comparisons at the item level only (Desa, 2014). This measurement issue also limits meaningful comparisons that could shed light on how teaching effectiveness relates to educational systems and teacher education in multilevel settings (Baumert et al., 2010; Scherer and Gustafsson, 2015).

Taken together, our review of substantive research revealed a number of challenges associated with the measurement of perceived instructional quality: First, individual students' perceptions of instructional quality are interwoven and may constitute general or specific evaluations of instruction. More precisely, given that the three factors of perceived instructional quality neither are clearly distinct with regards to their conceptualization nor with regards to their measurement, the assumption of perfect factor structures without any cross-loadings might be questionable. Moreover, general response tendencies may occur. Hence, accounting for cross-loadings on the one hand and distinguishing between a general factor capturing response tendencies and specific factors of perceived instructional quality on the other hand stresses the need for flexible, yet complex measurement models. Second, in comparative studies such as PISA, specific degrees of measurement invariance need to be ensured in order to compare factor means or correlations across countries. If invariance cannot be established, such comparisons may compromise the inferences researchers make on the basis of their results.

### Promising new modeling approaches

Psychometric research has identified at least two approaches that are capable of evaluating and potentially addressing the challenges of measuring perceived instructional quality: exploratory structural equation and bifactor modeling. Whereas exploratory structural equation modeling (ESEM) is a new modeling framework that specifically deals with cross-loadings (Marsh et al., 2009, 2014), bifactor modeling which is available both within the traditional CFA and the ESEM framework differentiates between general and specific factors of constructs (Chen et al., 2012; Reise, 2012). Both bifactor CFA and bifactor ESEM models can also be extended to multi-group models for invariance testing (Marsh et al., 2009; Chen et al., 2012). In the following, we will briefly outline these two modeling approaches and how they could be integrated into the measurement of perceived instructional quality (Table 1).

**Exploratory structural equation models** are a new family of latent variable models that combine exploratory factor analysis (EFA) with properties of CFA, allowing for the introduction of residual correlations, multi-group structures, or the incorporation of external variables to a factor model with cross-loadings (Asparouhov and Muthén, 2009; Marsh et al., 2009). Technically speaking, ESEM freely estimates all rotated cross-loadings that occur between items and factors (for details, please refer to Marsh et al., 2009). In a first step, the unconstrained factor structure is estimated. This preliminary structure is rotated in a second step by using a wide range of methods such as oblique or orthogonal rotations (for details, please refer to Asparouhov and Muthén, 2009). For instance, the oblique target rotation method assumes cross-loadings, which can be specified as being approximately zero. In the final model, however, these target loadings may result in values that significantly deviate from zero (Asparouhov and Muthén, 2009). The target rotation allows researchers to specify a-priori assumptions on the factor structure and can be regarded as an approximation of confirmatory factor analysis with approximately zero cross-loadings (Marsh et al., 2014). In contrast to exploratory factor analysis, ESEM allows researchers to test for measurement invariance across groups (e.g., countries; Dolan et al., 2009). Research has also shown that correlations between the factors and external variables are not overestimated in the ESEM approach (Asparouhov and Muthén, 2009). A more detailed description of ESEM and the rotation methods can be found in Marsh et al. (2014).

Unlike ESEM, **bifactor models** are not a new family of latent variable models in the sense that they introduce new methods of estimation. Rather, they are a recently more popular way of conceptualizing and parametrizing factor models by distinguishing between general and specific factors (Reise, 2012). These factors may explain variance in students' responses differently. This type of measurement model has experienced a recent advent, given that it provides a reasonable foundation for the conceptualization and measurement of psychological constructs in many substantive areas (e.g., Gustafsson and Åberg-Bengtsson, 2010; Chen et al., 2012; Wiesner and Schanding, 2013; Perera, 2015; Scherer et al., 2015; Stenling et al., 2015). In bifactor models, all items load on at least one first-order specific factor and on a general first-order factor that is orthogonal to all specific factors (Gignac and Watkins, 2013). They can be estimated both within the CFA and the ESEM framework. In common formulations of bifactor CFA models, cross-loadings between the specific factors are zero, reflecting the assumption of perfect item-factor links for the specific measurement part. However, recent methodological advances have extended bifactor CFA models to bifactor ESEM (Marsh et al., 2014). This combination of ESEM and bifactor modeling makes the features of both models available within a structural equation modeling framework (Morin et al., 2016). Generally speaking, in the context of evaluating students' perceptions of instructional quality, bifactor models may be particularly useful, because they allow researchers to control for general response tendencies by introducing a general factor (He and van de Vijver, 2013).

Linking the challenges associated with the modeling of individually perceived instructional quality with the recent methodological advances of ESEM and bifactor modeling, we argue that both approaches and their combination may provide powerful tools to evaluate students' perceptions with respect to their structure, invariance, and relations to external constructs.

### The present study

In light of our previous considerations, we aim at addressing the challenges of modeling individual students' perceptions of instructional quality on the basis of the PISA 2012 data. Moreover, we illustrate the application of different modeling approaches that describe the factor structure of these perceptions, and the information that can be obtained from them. In particular, we pose the following research questions:

1. To what extent can the structure of individual students' perceptions of instructional quality be described by different modeling approaches (CFA, ESEM, bifactor CFA, and bifactor ESEM)?
2. To what extent can measurement invariance across the three countries (i.e., Australia, Canada, and the USA) be supported?
3. To what extent are individual students' perceptions of instructional quality and educational outcomes (i.e., achievement, self-concept, and motivation) related?

To our knowledge, this study is the first to examine cross-country measurement invariance of individual students' perceived instructional quality by comparing different modeling approaches, and thereby testing the robustness of the results across methods. We take an individual differences perspective on the use of student ratings.

## Materials and methods

### Sample and procedure

We used the nationally representative PISA 2012 data sets of Australia (*n*_AUS_ = 9401 students in 773 schools), Canada (*n*_CAN_ = 14,057 students in 882 schools), and the United States of America (*n*_USA_ = 3288 students in 162 schools). These countries were selected for a number of reasons: First, as we were aimed at testing for measurement invariance across countries, we wanted to rule out language differences as causes for obvious deviations from invariance. Moreover, comparing these three countries with similar language and cultural backgrounds also adds to testing our findings and approaches for reproducibility across samples. As such, the choice of countries was driven by a robustness argument (Duncan et al., 2014). Second, these countries were chosen on the basis of previous studies that identified clusters of countries exhibiting similar profiles in mathematics achievement and related constructs (e.g., Marsh et al., 2013a). As such, the Anglo-Saxon cluster is one of the most robust clusters across studies (Kristen et al., 2005; Bulle, 2011). In the PISA 2012 database, these Anglo-Saxon countries provide large-scale and representative data with an exceptionally high psychometric quality (OECD, 2014b). A third reason for choosing these countries is that large-scale, quantitative studies on instructional quality have mostly been published on the basis of data obtained from German-speaking countries (e.g., Kunter et al., 2007; Lüdtke et al., 2009; Wagner et al., 2013; Fauth et al., 2014), whereas only few studies have been reported in the USA (e.g., Grossman et al., 2013; MET Project, 2013; Blazar, 2015); yet, many of these were qualitatively oriented, observational classrooms studies. Hence, our choice of the Anglo-Saxon countries adds to the body of studies on students' perceptions of instructional quality in these countries. Nevertheless, we point out that the samples chosen for the current study, more or less, represent examples with which a thorough investigation of these individual perceptions can be conducted.

The entire sample of students from the three countries who worked on the perceived instructional quality scale and took background questionnaires and achievement tests in mathematics, science, reading, and creative problem solving, comprised *N* = 26,746 students (50.0% female), aged between 15.25 and 16.33 years (*M* = 15.82, *SD* = 0.29 years). Both, the questionnaires and achievement tests were designed in such a way that each student had to work on only a selected number of items (rotated incomplete block design; OECD, 2013b). The procedures of test administration, coding of responses, and data preparation were employed according to the PISA 2012 quality standards (OECD, 2014a,b). Ethics approval for the PISA 2012 study was granted by the OECD Board of Participating Countries (BPC).

### Measures

In order to address our research questions, we used students' responses on their perceived teacher support, cognitive activation, and classroom management. Students had to rate a number of statements on frequency-based, four-point scales ranging from 0 (*never or hardly ever*) to 3 (*every lesson*). These statements and their descriptive statistics are shown in Supplementary Tables S1, S2. We estimated McDonald's ω as a measure of scale reliability (see Table 2; Yang and Green, 2011).

**Table 2:** **Reliabilities of the scales measuring perceived instructional quality, self-concept, and motivation (reported as McDonald's ω)**.

**Scale**	**Australia**	**Canada**	**USA**	**Total sample**
Teacher support	0.90	0.87	0.87	0.87
Cognitive activation	0.88	0.86	0.87	0.87
Classroom management	0.91	0.88	0.89	0.89
Self-concept	0.88	0.90	0.89	0.89
Intrinsic motivation	0.91	0.91	0.91	0.91
Instrumental motivation	0.92	0.91	0.92	0.92

#### Teacher support

Teacher support was measured by 5 items that referred to teachers' interest in students' learning as manifested in the provision of helpful and supportive opportunities-to-learn (Klusmann et al., 2008; Klieme et al., 2009). This scale showed high reliabilities between ω = 0.87 and ω = 0.90 (Table 2).

#### Cognitive activation

The scale on teachers' attempts to activate students cognitively comprised 9 items, which focused on the provision of challenging tasks, the application of prior knowledge, and the evaluation and exploration of problem solutions (Wagner et al., 2013; Fauth et al., 2014). This scale showed high reliabilities between ω = 0.86 and ω = 0.87 (Table 2).

#### Classroom management

Students' perceptions of classroom management were assessed by 5 items, which covered aspects of how teachers cope with disruptions and how they create an environment of order and discipline in the classroom (e.g., “*Students don't start working for a long time after the lesson begins*.”). These aspects have been identified in numerous studies and research indicated the validity of this conceptualization (Seidel and Shavelson, 2007; OECD, 2013b; Wagner et al., 2013). The scale showed high reliabilities between ω = 0.88 and ω = 0.91 (Table 2).

#### Achievement in mathematics

PISA 2012 conceptualized students' mathematical literacy as a construct that comprises mathematical processes (i.e., Formulating situations mathematically; employing mathematical concepts, facts, procedures, and reasoning; interpreting, applying, and evaluating mathematical outcomes) and content categories (i.e., change and relationships; space and shape; quantity; uncertainty and data; OECD, 2013b, 2014a). Since 109 mathematics items were used to assess mathematical literacy, a rotated-booklet design was implemented to reduce the number of items a single student had to work on. In consequence, PISA 2012 provided a set of five plausible values for mathematics achievement that reflect students' performance (Wu, 2005; OECD, 2014b). For more details on the plausible value technique applied in PISA 2012, please refer to OECD (2014b). The reliabilities of the achievement test ranged between 0.92 and 0.94 (OECD, 2014b, p. 234).

#### Self-concept in mathematics

Students' self-concept in mathematics refers to their beliefs in their abilities in mathematics and can be regarded as an important predictor of student achievement (Marsh, 1986; Marsh and Martin, 2011). In our secondary analyses, we chose the four positively worded items that were available in PISA 2012. Students had to rate them on a four-point Likert scale ranging from 0 (“*I strongly disagree*”) to 3 (“*I strongly agree*”). This scale showed high reliabilities between ω = 0.88 and ω = 0.90 (Table 2).

#### Intrinsic and instrumental motivation to learn mathematics

PISA 2012 distinguishes between two aspects of motivation which are considered to be key constructs in common theories on motivation (Eccles and Wigfield, 2002). First, *students' intrinsic motivation to learn mathematics* is defined as students' “drive to perform an activity purely for the joy gained from the activity itself” (OECD, 2013a, p. 65). This construct was assessed by 4 items (e.g., “*I do mathematics because I enjoy it*”), which students had to rate on a four-point Likert scale (from 0 = “*I strongly disagree*” to 3 = “*I strongly agree*”). Second, *students' instrumental motivation* to learn mathematics refers to the “drive to learn mathematics because students perceive it as useful to them and to their future studies and careers” (OECD, 2013a, p. 70). Based on the same Likert scale, the construct was assessed by 4 items such as “*Learning mathematics is worthwhile for me because it will improve my career prospects and chances*.” Both scales showed high reliabilities between ω = 0.91 and ω = 0.92 (Table 2).

Students' self-concept, intrinsic, and instrumental motivation are considered to be desirable educational outcomes that are positively and significantly linked to achievement in mathematics (Marsh and Martin, 2011; Murayama et al., 2013). In fact, educational psychologists argue that these constructs are among the most important predictors of achievement and students' future career aspirations (e.g., Parker et al., 2014). In our study, we use them as a nomological network to study how different approaches to modeling students' perceptions of instructional quality affect its relations to other constructs.

### Statistical analyses

#### Measurement models and model fit

**Figure 1:**
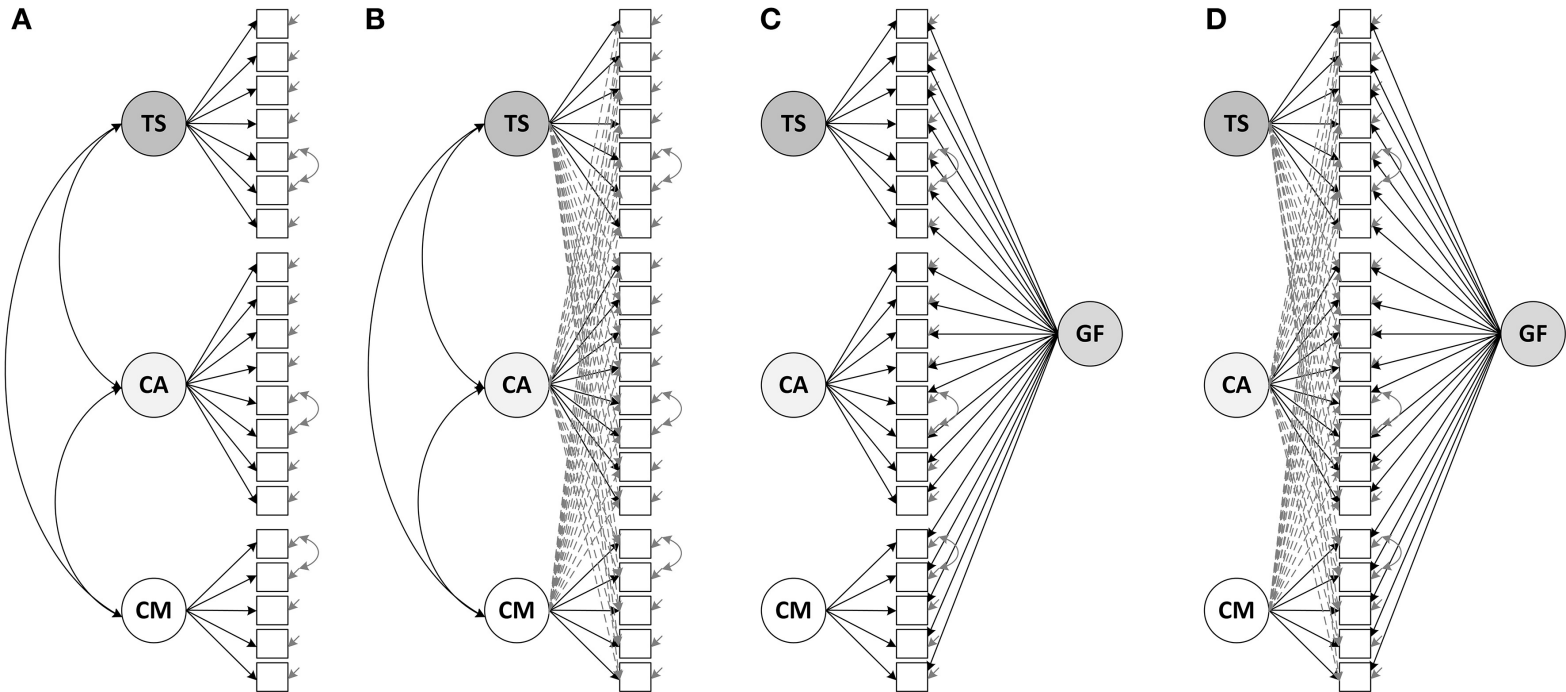
**Measurement models of students' perceived instructional quality: (A) CFA model without cross-loadings, (B) ESEM with cross-loadings, (C) Bifactor CFA model, (D) Bifactor ESEM with cross-loadings**. TS, Teacher support; CA, Cognitive activation; CM, Classroom management; GF, General factor of students' perceptions.

In order to address Research Question 1, we tested different measurement models of students' perceptions. Each of these models reflects different assumptions on the factor structure (see Figure 1). First, we tested whether the theoretically implied, three-factor structure held, using confirmatory factor analysis without cross-loadings. This approach assumes a simple structure of the data, that is, a perfect link between items and factors. Second, we specified an ESEM with oblique target rotation, allowing for cross-loadings such that each item may load on each of the three factors. Third, we specified a bifactor CFA model, assuming a general factor of students' perceptions and three specific factors which capture the unique variances in the item responses for the three aspects of instructional quality. Fourth, we extended the bifactor CFA model by introducing cross-loadings. For specifying this bifactor ESEM approach, we constrained the factor correlations to zero, using orthogonal target rotation (Morin et al., 2016; see Supplementary Figure S1). An overview of each model's properties with regard to cross-loadings and generality/specificity of factors can be found in Table 1 (also see Figure 1 for a graphical depiction of the models).

In order to evaluate the model fit, we referred to common guidelines (CFI ≥ 0.95, TLI ≥ 0.95, RMSEA ≤ 0.08, SRMR ≤ 0.10 for an acceptable model fit; Marsh et al., 2005). For model comparisons, we evaluated the differences in CFI, TLI, RMSEA, and SRMR, and, additionally, the differences in information criteria such as Akaike's and the Bayesian Information Criterion (AIC, BIC).

Robust maximum likelihood estimation (MLR) with standard errors and tests of fit that were robust against non-normality of item responses and the use of categorical variables in the presence of at least four response categories was used (Rhemtulla et al., 2012). In all models using the MLR estimation, we treated the item responses continuously^1^. We selected this method over the weighted least squares means and variance adjusted (WLSMV) estimation for three main reasons (for a similar reasoning, please refer to Aguado et al., 2015): First, students' responses were coded with at least four response categories on a frequency scale (Beauducel and Herzberg, 2006; Rhemtulla et al., 2012). Second, in contrast to the WLSMV estimation, this procedure provides robust results even in the presence of missing data that follow the “missing at random” mechanism (Asparouhov and Muthén, 2010). Third, we followed the recommendations given by Marsh et al. (2009) for using the MLR estimation and continuous treatment of student responses despite their categorical nature for using ESEM in order to model the factor structure, measurement invariance, and the relations to further constructs (see also Marsh et al., 2013b). In this sense, we provide comparability with these previous studies with respect to the application of ESEM generally (e.g., Marsh et al., 2009; Chung et al., 2015; Stenling et al., 2015) and the application of bifactor ESEM specifically (Morin et al., 2016).

All analyses were conducted in the statistical program M*plus* 7.3 (Muthén and Muthén, 1998–2014).

#### Measurement invariance testing

To address Research Question 2, different steps of measurement invariance testing were taken, using multi-group modeling. We tested the measurement models obtained from the results on Research Question 1 for configural, metric, scalar, and strict invariance by systematically constraining factor loadings, item intercepts, and item uniquenesses (i.e., item-specific residual variances) to equality across countries (Marsh et al., 2009; Millsap, 2011; van de Schoot et al., 2013): *Configural invariance* is established when the numbers of factors and the factor loading patterns (i.e., the items are assumed to load on the same factors in all groups) are the same across countries. In a configural invariance model, factor loadings, item intercepts, and item uniquenesses are freely estimated in each country. *Metric invariance* is established by constraining the factor loadings in the configural model and thereby putting the latent factors on the same scale. This level is required for comparisons of the relations to external variables. The *scalar invariance* model further constrains the item intercepts. This model forms the prerequisite for comparing factor means across countries (Millsap, 2011). Finally, *strict invariance* is established when the item uniquenesses are also constrained; facilitating comparisons among the means of manifest variables (e.g., sum scores). These invariance models were specified for each of the four measurement models (i.e., CFA, ESEM, bifactor CFA, and bifactor ESEM). For the ESEM approach, main and cross-loadings were constrained in the cases of metric, scalar, and strict invariance (see Supplementary Figure S2).

We evaluated the invariance models on the basis of their goodness-of-fit and the results from the model comparisons. However, regarding the model fit, we did not rely on χ^2^ difference testing for interpreting the fit of nested models, because the χ^2^ statistic strongly depends on the sample size and is overly sensitive to even trivial misfit (Little, 2013). Instead, we followed the recommendations given by Cheung and Rensvold (2002) and Rutkowski and Svetina (2014), and considered the changes of the incremental fit indices as practically insignificant if the CFI and TLI decreased less than 0.010, and the RMSEA and SRMR decreased less than 0.015, compared to the configural model. These statistics are particularly sensitive to deviations from invariance of factor loadings, intercepts, and uniquenesses (Chen, 2007). Nevertheless, it is currently unclear to what extent the information criteria (AIC, BIC) are sensitive toward the equality constraints across groups. In fact, to our knowledge, there have been no specific recommendations on potential cut-offs or “rules of thumb” for the use of ΔAIC and ΔBIC. This particularly applies to the context of testing measurement invariance in complex international large-scale data sets with large samples sizes in the presence of missing values. As a consequence, we have put more emphasis on the changes in CFI, TLI, RMSEA, and SRMR when evaluating the effect of equality constraints. Specifically, if these changes were within the suggested guidelines and, in addition, the absolute model fit was acceptable, we interpreted this findings as evidence for measurement invariance, although the AIC and BIC may have increased. Finally, we followed the common CFA and ESEM invariance testing approach that was proposed by Marsh et al. (2013b).

We note that ignoring non-invariance may lead to biased estimates of parameters in the measurement model and the regression coefficients to external variables (Guenole and Brown, 2014).

In the cases where at least metric invariance was met, we addressed Research Question 3. Specifically, we extended the multi-group models by adding structural relations to students' achievement, self-concept, and motivation as educational outcomes. Hence, for these models, we investigated whether the relations to educational outcomes differed across the modeling approaches.

#### Selection bias, hierarchical structure, and missing data

In PISA 2012, students and schools were randomly sampled in a two-stage procedure within countries (OECD, 2014b). Due to different probabilities of being selected as a student to participate in the large-scale study, sampling error may occur. We therefore used students' final weights (FSTUWT) in all analyses to correct for potential selection bias (Asparouhov, 2005). Moreover, given that students are nested within schools, we adjusted the standard errors and χ^2^ statistics in all models (M*plus* option TYPE = COMPLEX). The resulting χ^2^ statistics were therefore Satorra-Bentler corrected (SB-χ^2^; Satorra and Bentler, 2010).

Among the students who took the questionnaire on their perceptions of instructional quality, self-concept, and motivation, 1.4% missing values occurred. Given that these missing values were not due to the design of the study, we assumed that they occurred randomly and consequently applied the full-information-maximum-likelihood (FIML) procedure as a model-based treatment of missing data (Enders, 2010).

All analyses that involved the achievement scores were conducted with each of the five plausible values separately and the resulting model parameters (e.g., regression coefficients) were combined using the TYPE = IMPUTATION option in M*plus* (von Davier et al., 2009). This option combines the model parameters across the five data sets (each of which contains one plausible value) according to Rubin's rules (Enders, 2010).

## Results

### Research question 1: measurement models of perceived instructional quality

We evaluated the different assumptions on the factor structure of students' perceptions of instructional quality to approach Research Question 1.

As a first step, we tested whether a unidimensional factor model provided an appropriate model fit for the total sample, assuming that students' perceptions reflect a unitary construct. This model resulted in an overall poor fit, SB-$\chi_{\left(149\right)}^{2}=$ 10,640.0, *p* < 0.001, CFI = 0.618, TLI = 0.562, RMSEA = 0.051 (0.050, 0.052), SRMR = 0.141, AIC = 1,166,014, BIC = 1,166,506, and was therefore rejected.

As a second step, we tested a CFA model with three correlated factors but without cross-loadings. This model resulted in an acceptable goodness-of-fit, as shown for the total sample in Table 3. This finding provides some evidence for the distinction between the three hypothesized factors of perceived instructional quality.

**Table 3:** **Fit statistics of measurement models**.

**Model**	**SB-χ^2^ (df)**	**CFI**	**TLI**	**RMSEA (90%-CI)**	**SRMR**	**AIC**	**BIC**
**AUSTRALIAN SAMPLE (*N* = 9401)**							
CFA	2354.3 (146)	0.964	0.958	0.040 (0.039, 0.042)	0.039	377,418	377,868
ESEM	1259.8 (114)	0.981	0.972	0.033 (0.031, 0.034)	0.016	375,802	376,481
Bifactor CFA	1201.1 (130)	0.983	0.977	0.030 (0.028, 0.031)	0.026	375,625	376,190
Bifactor ESEM	755.9 (98)	0.989	0.981	0.027 (0.025, 0.029)	0.011	374,998	375,791
**CANADIAN SAMPLE (*N* = 14,057)**							
CFA	2158.4 (146)	0.955	0.947	0.031 (0.030, 0.032)	0.039	577,287	577,763
ESEM	1273.9 (114)	0.974	0.961	0.027 (0.026, 0.028)	0.019	575,045	575,762
Bifactor CFA	1190.3 (130)	0.976	0.969	0.024 (0.023, 0.025)	0.030	574,697	575,294
Bifactor ESEM	775.3 (98)	0.985	0.973	0.022 (0.021, 0.024)	0.013	573,686	574,524
**US-AMERICAN SAMPLE (*N* = 3288)**							
CFA	1009.4 (146)	0.957	0.950	0.042 (0.040, 0.045)	0.037	133,720	134,104
ESEM	739.6 (114)	0.969	0.953	0.041 (0.038, 0.044)	0.022	133,422	134,001
Bifactor CFA	668.9 (130)	0.973	0.965	0.036 (0.033, 0.038)	0.047	133,256	133,738
Bifactor ESEM	435.2 (98)	0.983	0.971	0.032 (0.029, 0.035)	0.014	132,995	133,672
**TOTAL SAMPLE (*N* = 26, 746)**							
CFA	1307.5 (146)	0.958	0.951	0.017 (0.016, 0.018)	0.037	1,088,470	1,088,986
ESEM	927.8 (114)	0.970	0.956	0.016 (0.015, 0.017)	0.021	1,085,465	1,086,243
Bifactor CFA	834.9 (130)	0.974	0.966	0.014 (0.013, 0.015)	0.044	1,084,405	1,085,052
Bifactor ESEM	532.4 (98)	0.984	0.972	0.013 (0.012, 0.014)	0.013	1,081,998	1,082,907

*SB-χ^2^ (df), Satorra-Bentler corrected χ^2^value with df degrees of freedom. SB-χ^2^ values are statistically significant at the 0.1% level. 90%-CI, 90% confidence interval of the RMSEA*.

As a third step, we specified a three-factor ESEM as a more flexible measurement model that loosens the assumption of zero cross-loadings. This model showed a good fit (see Table 3, Total sample, ESEM) and, furthermore, outperformed the CFA approach: ΔCFI = +0.012, ΔTLI = +0.005, ΔRMSEA = −0.001, ΔSRMR = −0.016, ΔAIC = −3005, ΔBIC = −2743. Looking at the factor loadings for both the CFA and the ESEM approach, we observed that the main loadings of the instructional quality items were rather high for the factors the items were originally assigned to (see Table 4). In addition, for selected items such as ST80Q08 (*The teacher helps us to learn from mistakes we have made*.), significant cross-loadings occurred. But given that these cross-loadings were comparably small (|λ| ≤ 0.22), their effect on the factor correlations were marginal. In this respect, the highest correlation among the three factors occurred between teacher support and cognitive activation for both modeling approaches. Still, the link between items and factors was not perfect, given that an overlap between the factors beyond factor correlations existed. Hence, the assumptions on the factor structure in the CFA approach may not fully represent the nature of students' perceptions.

**Table 4:** **Factor loadings and correlations obtained from the different measurement models for the total sample**.

	**CFA/ESEM**			**Bifactor CFA/ESEM**			
	**Teacher support**	**Cognitive activation**	**Classroom management**	**General factor**	**Teacher support**	**Cognitive activation**	**Classroom management**
**FACTOR LOADINGS**							
ST77Q01	0.73^*^/0.68^*^	0.06^*^	0.04	0.51^*^/0.52^*^	0.51^*^/0.49^*^	0.04^*^	0.09^*^
ST77Q02	0.76^*^/0.74^*^	0.02	0.01	0.52^*^/0.55^*^	0.55^*^/0.51^*^	–0.01	0.07^*^
ST77Q03	0.79^*^/0.79^*^	0.00	0.01	0.53^*^/0.57^*^	0.59^*^/0.54^*^	–0.03	0.07^*^
ST77Q04	0.80^*^/0.85^*^	–0.04^*^	–0.03	0.51^*^/0.54^*^	0.64^*^/0.61^*^	–0.01	0.05^*^
ST77Q05	0.70^*^/0.65^*^	0.06^*^	0.03	0.48^*^/0.47^*^	0.51^*^/0.51^*^	0.09^*^	0.09^*^
ST80Q01	0.05	0.72^*^/0.69^*^	0.01	0.62^*^/0.58^*^	0.05^*^	0.37^*^/0.42^*^	–0.02
ST80Q04	–0.12^*^	0.65^*^/0.74^*^	0.02	0.44^*^/0.41^*^	0.02	0.62^*^/0.61^*^	0.00
ST80Q05	–0.04	0.56^*^/0.62^*^	–0.06^*^	0.41^*^/35^*^	0.08^*^	0.48^*^/0.56^*^	–0.07^*^
ST80Q06	–0.15^*^	0.47^*^/0.58^*^	–0.03	0.27^*^/0.23^*^	0.02	0.54^*^/0.57^*^	–0.04^*^
ST80Q07	0.02	0.71^*^/0.70^*^	0.02	0.62^*^/0.59^*^	0.00	0.34^*^/0.39^*^	–0.02
ST80Q08	0.22^*^	0.72^*^/0.57^*^	0.00	0.81^*^/0.77^*^	0.02	**0.04**/0.13^*^	–0.05^*^
ST80Q09	0.06	0.65^*^/0.61^*^	–0.01	0.64^*^/0.66^*^	–0.10^*^	0.17^*^/0.17^*^	–0.07^*^
ST80Q10	0.01	0.70^*^/0.69^*^	0.04	0.64^*^/0.65^*^	–0.07^*^	0.27^*^/0.28^*^	–0.02
ST80Q11	–0.01	0.65^*^/0.66^*^	–0.01	0.56^*^/0.55^*^	–0.04	0.30^*^/0.33^*^	–0.06^*^
ST81Q01	0.02	–0.01	0.75^*^/0.75^*^	0.17^*^/0.19^*^	0.07^*^	–0.03	0.73^*^/0.72^*^
ST81Q02	–0.07^*^	0.03	0.81^*^/0.83^*^	0.16^*^/0.19^*^	0.01	–0.01	0.80^*^/0.79^*^
ST81Q03	–0.02	–0.03	0.84^*^/0.86^*^	0.14^*^/0.17^*^	0.05^*^	–0.04^*^	0.84^*^/0.83^*^
ST81Q04	0.07^*^	0.00	0.75^*^/0.73^*^	0.22^*^/0.25^*^	0.09^*^	–0.04	0.72^*^/0.70^*^
ST81Q05	0.02	0.01	0.76^*^/0.75^*^	0.19^*^/0.23^*^	0.05^*^	–0.05^*^	0.73^*^/0.72^*^
**FACTOR CORRELATIONS**							
Teacher support				0.00/0.00			
Cognitive activation	0.60^*^/0.56^*^			0.00/0.00	0.00/0.00		
Classroom management	0.31^*^/0.32^*^	0.14^*^/0.13^*^		0.00/0.00	0.00/0.00	0.00/0.00	

*The table shows the fully standardized estimates. Parameters of the CFA models are shown in bold and appear before the forward stash*.

* *p < 0.01*.

As a fourth step, we estimated a bifactor CFA model, distinguishing between general and specific variance components in students' perceptions. This model showed a good model fit to the data of the total sample (see Table 3, Bifactor CFA Model), and outperformed both the CFA (ΔCFI = +0.016, ΔTLI = +0.015, ΔRMSEA = −0.003, ΔSRMR = +0.007, ΔAIC = −4065, ΔBIC = −3934) and the ESEM approaches (ΔCFI = +0.004, ΔTLI = +0.010, ΔRMSEA = −0.002, ΔSRMR = +0.023, ΔAIC = −1060, ΔBIC = −1191). Regarding the model structure, significant factor loadings on the general factor and the specific factors were observed, pointing to the general and specific variance components in item responses (see Table 4, Bifactor CFA). Interestingly, items that were assigned to the factor of perceived classroom management (ST81Q01–ST81Q05) showed high loadings on the specific but low loadings on the general factor.

Finally, in our fifth step, we applied bifactor modeling to the ESEM approach. The resulting bifactor ESEM fitted the data of the total sample very well (Table 3, Bifactor ESEM). Furthermore, comparing this model to the CFA (ΔCFI = +0.026, ΔTLI = +0.021, ΔRMSEA = −0.004, ΔSRMR = −0.024, ΔAIC = −6472, ΔBIC = −6079), ESEM (ΔCFI = +0.014, ΔTLI = +0.016, ΔRMSEA = −0.003, ΔSRMR = −0.008, ΔAIC = −3467, ΔBIC = −3336), and bifactor CFA modeling (ΔCFI = +0.010, ΔTLI = +0.006, ΔRMSEA = −0.001, ΔSRMR = −0.031, ΔAIC = −2407, ΔBIC = −2145) suggested its superiority in model fit. As for the bifactor CFA model, all items loaded positively on the general factor; the highest loadings for the classroom management factor were, however, observed on the specific factor with the loadings on the general factor being rather low compared to the other two facets of student-perceived instructional quality (see Table 4, Bifactor ESEM). Furthermore, cross-loadings in the specific part of the model were rather low (|λ| ≤ 0.10), since the main loadings occurred on the specific factors, the items were originally assigned to.

Our findings on the performance of the three types of measurement models were completely replicated for each of the three countries separately (see Table 3). Replicating the results from the total sample, the CFA approach performed worse than the ESEM and bifactor approaches. The bifactor ESEM served as the most appropriate representation of individual students' perceptions of instructional quality.

### Research question 2: measurement invariance across countries

On the basis of the different measurement models that describe the structure of students' perceived instructional quality, we examined their measurement invariance across the three countries (Research Question 2).

As shown in Table 5, the changes in model fit (CFI, RMSEA, and SRMR) were below the suggested cut-off values (Cheung and Rensvold, 2002). Moreover, each of the four measurement models fitted the data reasonably well at all levels of measurement invariance. That is, even though the models clearly have different assumptions with regard to the factor structure, measurement invariance of the parameters within each modeling approach seems to hold. As a consequence, comparisons of the relations to other constructs and the means of latent and manifest variables are meaningful, given that the measurement model has the same properties (i.e., number of factors, factor loadings, intercepts, and uniquenesses) in the three countries. Accordingly, the changes in model fit and the acceptable goodness-of-fit statistics provided evidence for strict invariance for the CFA, ESEM, bifactor CFA, and the bifactor ESEM approaches (Table 5). Again, it is noteworthy that the overall fit of the bifactor ESEM invariance models was superior in comparison to the other approaches. In sum, the four types of models can be used for cross-country comparisons. Please find these comparisons of factor means in the Supplementary Table S3.

**Table 5:** **Results on measurement invariance testing of the different modeling approaches**.

**Model**	**SB-χ^2^ (df)**	**CFI**	**TLI**	**RMSEA (90%-CI)**	**SRMR**	**AIC**	**BIC**	**ΔCFI/ΔTLI**	**ΔRMSEA/ΔSRMR**
**CFA**									
Configural	5788.7 (438)	0.959	0.952	0.037 (0.036, 0.038)	0.039	1,088,425	1,089,973	–	–
Metric	5962.5 (470)	0.958	0.954	0.036 (0.035, 0.037)	0.040	1,088,521	1,089,808	–0.001/+0.002	–0.001/+0.001
Scalar	6834.5 (502)	0.951	0.950	0.038 (0.037, 0.038)	0.042	1,090,006	1,091,030	–0.008/–0.002	+0.001/+0.003
Strict	7071.6 (540)	0.950	0.952	0.037 (0.036, 0.038)	0.044	1,090,401	1,091,114	–0.009/0.000	0.000/+0.005
**ESEM**									
Configural	3413.9 (342)	0.976	0.964	0.032 (0.031, 0.033)	0.019	1,084,268	1,086,604	–	–
Metric	3731.0 (438)	0.975	0.970	0.029 (0.028, 0.030)	0.021	1,084,456	1,084,995	–0.001/+0.006	–0.003/+0.002
Scalar	4545.0 (470)	0.968	0.966	0.031 (0.030, 0.032)	0.025	1,085,830	1,087,116	–0.008/+0.002	–0.001/+0.006
Strict	4803.4 (508)	0.967	0.966	0.031 (0.030, 0.032)	0.028	1,086,247	1,087,223	–0.009/+0.002	–0.001/+0.009
**BIFACTOR CFA**									
Configural	3196.4 (390)	0.978	0.971	0.028 (0.028, 0.029)	0.031	1,083,579	1,085,521	–	–
Metric	3462.3 (458)	0.977	0.974	0.027 (0.026, 0.028)	0.038	1,083,890	1,085,275	–0.001/+0.003	–0.001/+0.007
Scalar	4270.1 (488)	0.971	0.969	0.029 (0.029, 0.030)	0.039	1,085,258	1,086,397	–0.007/–0.002	+0.001/+0.008
Strict	4526.5 (526)	0.969	0.970	0.029 (0.028, 0.030)	0.041	1,085,674	1,086,502	–0.009/–0.001	+0.001/+0.010
**BIFACTOR ESEM**									
Configural	2055.7 (294)	0.986	0.976	0.026 (0.025, 0.027)	0.012	1,081,678	1,084,407	–	–
Metric^#^	3001.7 (407)	0.980	0.975	0.026 (0.025, 0.027)	0.017	1,083,028	1,084,748	–0.006/–0.001	0.000/+0.005
Scalar	3159.7 (444)	0.979	0.976	0.026 (0.025, 0.027)	0.020	1,083,272	1,084,772	–0.007/0.000	0.000/+0.008
Strict	3407.2 (482)	0.977	0.976	0.026 (0.025, 0.027)	0.023	1,083,668	1,084,856	–0.009/0.000	0.000/+0.011

*SB-χ^2^ (df), Satorra-Bentler corrected χ^2^ value with df degrees of freedom. SB-χ^2^ values are statistically significant at the 0.1% level. 90%-CI, 90% confidence interval of the RMSEA. Model comparisons refer to the configural invariance model as the baseline*.

# *To identify the metric model, one residual correlation had to be removed (items ST77Q02–ST77Q04)*.

### Research question 3: the relations between perceived instructional quality and educational outcomes

Given that even strict invariance could be established, we were able to compare the relations among students' perceptions of instructional quality and educational outcomes across countries (Research Question 3). These relations were estimated in structural equation models, in which all educational outcomes were regressed on the three factors of perceived instructional quality simultaneously. The results are reported in Table 6. Again, we examined these relations for each of the four modeling approaches.

**Table 6:** **Relations between students' perceptions of instructional quality (independent variables) and educational outcomes (dependent variables)**.

**β**	**CFA/ESEM**			**Bifactor CFA/ESEM**			
	**Teacher support**	**Cognitive activation**	**Classroom management**	**General factor**	**Teacher support**	**Cognitive activation**	**Classroom management**
**AUSTRALIAN SAMPLE**							
Achievement	0.01/–0.01	0.06^**^/0.07^**^	0.31^**^/0.32^**^	0.10^**^/0.16^**^	0.07^**^/0.04	0.11^**^/0.07^**^	0.31^**^/0.30^**^
Self-concept	0.10^**^/0.11^**^	0.22^**^/0.21^**^	0.15^**^/0.15^**^	0.30^**^/0.32^**^	0.07^**^/0.07^**^	0.12^**^/0.11*	0.14^**^/0.14^**^
Intrinsic motivation	0.16^**^/0.18^**^	0.25^**^/0.24^**^	0.11^*^/0.11^**^	0.39^**^/40^**^	0.07^**^/0.09^**^	0.09^**^/0.11^**^	0.10^**^/0.10^**^
Instrumental motivation	0.15^**^/0.17^**^	0.22^**^/0.21^**^	0.09^**^/0.10^**^	0.36^**^/37^**^	0.05/0.06^*^	0.06^*^/0.07^**^	0.08^**^/0.08^**^
**CANADIAN SAMPLE**							
Achievement	0.04/0.03	0.03/0.04	0.20^**^/0.20^**^	0.08^**^/0.13^**^	0.08^**^/0.04	0.07^**^/0.03	0.20^**^/0.20^**^
Self-concept	0.16^**^/0.17^**^	0.12^**^/0.11^**^	0.08^**^/0.08^**^	0.27^**^/0.28^**^	0.08^**^/0.10^**^	0.04^*^/0.05^**^	0.07^**^/0.07^**^
Intrinsic motivation	0.13^**^/0.15^**^	0.20^**^/0.19^**^	0.11^**^/0.12^**^	0.35^**^/0.35^**^	0.05/0.08^**^	0.06^*^/0.09^**^	0.10^**^/0.10^**^
Instrumental motivation	0.14^**^/0.15^**^	0.18^**^/0.17^**^	0.08^**^/0.09^**^	0.29^**^/0.31^**^	0.09^**^/0.10^**^	0.09^**^/0.09^**^	0.08^**^/0.08^**^
**US-AMERICAN SAMPLE**							
Achievement	0.01/0.00	0.02/0.04	0.30^**^/0.30^**^	0.07^**^/0.13^**^	0.07^*^/0.04	0.09^**^/0.05	0.30^**^/0.30^**^
Self-concept	0.17^**^/0.18^**^	0.14^**^/0.14^**^	0.11^**^/0.12^**^	0.30^**^/0.31^**^	0.12^**^/0.13^**^	0.07^**^/0.08^**^	0.11^**^/0.11^**^
Intrinsic motivation	0.18^**^/0.20^**^	0.19^**^/0.18^**^	0.09^**^/0.09^**^	0.36^**^/0.34^**^	0.10^**^/0.14^**^	0.07^*^/0.11^**^	0.08^**^/0.08^**^
Instrumental motivation	0.17^**^/0.19^**^	0.22^**^/0.21^**^	0.05/0.05	0.36^**^/0.36^**^	0.09^**^/0.12^**^	0.08^*^/0.10^**^	0.04/0.04

*The table shows the fully standardized regression coefficients from a multivariate regression model. Parameters of the CFA model appear before the forward stash*.

* *p < 0.05*,

** *p < 0.01*.

In particular, for the CFA and ESEM, extending the measurement models by introducing the educational outcome variables led to acceptable model fits, CFA: SB-$\chi_{\left(1,\;461\right)}^{2}=$ 11,900.6, *p* < 0.001, CFI = 0.953, TLI = 0.952, RMSEA = 0.028, SRMR = 0.035, AIC = 1,806,666, BIC = 1,808,460; ESEM: SB-$\chi_{\left(1,\;429\right)}^{2}=9528.0$, *p* < 0.001, CFI = 0.964, TLI = 0.962, RMSEA = 0.025, SRMR = 0.028, AIC = 1802,492, BIC = 1,804,549. These models revealed positive relations among the three instructional variables, achievement, self-concept, and motivation for each country. The resulting relations did not differ significantly between the two modeling approaches, strengthening their robustness across methods. It is noteworthy that the highest regression coefficients for the motivational constructs could be identified for perceived teacher support, and cognitive activation (β = 0.12–0.25), whereas the relations to classroom management were generally lower (β = 0.05–0.15). Moreover, we could not find any significant differences in the relations between countries, pointing to their cross-country robustness. Students' achievement could be best predicted by perceived classroom management (β = 0.20–0.31), yet not by teacher support for all three countries. For the Australian sample, the regression coefficients were significant but low for cognitive activation (β = 0.06–0.07), whereas there was not significant relation in the other samples.

In this regard, we would like to point out that using the highly correlated factors of perceived instructional quality as predictors of educational outcomes needs to be taken with caution, because multicollinearity may compromise the resulting regression coefficients. However, this problem does not occur in the bifactor models, which assume uncorrelated factors (Reise, 2012).

The extended bifactor approaches fitted the data well [Bifactor CFA: SB-$\chi_{\left(1,\;437\right)}^{2}=$ 9205.3, *p* < 0.001, CFI = 0.965, TLI = 0.964, RMSEA = 0.025, SRMR = 0.037, AIC = 1,801,889, BIC = 1,803,880; Bifactor ESEM: SB-$\chi_{\left(1,\;391\right)}^{2}=$ 8070.7, *p* < 0.001, CFI = 0.970, TLI = 0.968, RMSEA = 0.023, SRMR = 0.026, AIC = 1,799,900, BIC = 1,802,268], and unraveled positive and strong associations between the general factor, the motivational constructs (β = 0.29–0.40), and self-concept (β = 0.27–0.32). The specific factors, however, correlated only slightly with self-concept and motivation (Table 6). Regarding students' achievement, most of the variance could be explained by perceived classroom management (β = 0.20–0.31). In contrast to self-concept and motivation, the general factor was only marginally related to mathematical literacy (β = 0.07–0.16). Again, the two different bifactor approaches indicated the same tendencies of relations to educational outcomes. Still, some differences occurred in the relations to students' achievement. For instance, the specific factor of perceived teacher support was not significantly associated with achievement for the bifactor ESEM in all countries, whereas the bifactor CFA approach revealed low but significant regression coefficients (β = 0.07–0.08). In this respect, there is a slight effect of accounting for cross-loadings in these relations.

Taken together, we identified positive relations between students' perceptions of instructional quality, their achievement, self-concept, and motivation in mathematics across countries. These findings were, by and large, robust against the different modeling approaches.

## Discussion

The main goal of the present study was to compare different factor models of students' perceived instructional quality with respect to their structure, measurement invariance, and relations to educational outcomes. Making use of a large-scale data set obtained from PISA 2012, we introduced four modeling approaches that reflect different assumptions about the structure of students' perceptions of instructional quality, and showed that measurement invariance across three selected countries could be established. Extending the measurement models suggested the persistence of three factors of perceived instructional quality and significant relations to cognitive and motivational outcomes. We could show that, using these new approaches (more specifically, bifactor ESEM), it is possible to both account for item cross-loadings and to disentangle general and specific factors of students' perceptions.

Overall, we found that all four modeling approaches including CFA showed sufficient properties such as acceptable model fit and levels of measurement invariance. However, all three more complex modeling approaches (ESEM, bifactor CFA, bifactor ESEM) outperformed the traditional CFA approach with regard to model fit, and did not show any disadvantages in invariance tests. Furthermore, specific relations to educational outcomes for each of the facets of instructional quality seem to be better captured using the bifactor approaches.

### Measurement models of perceived instructional quality and their invariance

The results on the factor structure of individual students' perceptions of instructional quality are in line with previous research and strengthen the distinction between perceived teacher support, cognitive activation, and classroom management (Klieme, 2013; Wagner et al., 2013; Fauth et al., 2014). These results show that students are generally able to distinguish between the three factors of instructional quality in their ratings. However, this distinction is not perfect, as significant cross-loadings between cognitive activation and teacher support indicated (up to λ = 0.22). We argue that this is not merely a measurement issue, but it reflects a conceptual overlap in the substantive definitions of the facets of instructional quality.

As a consequence, we took a novel perspective on students' perceived instructional quality by proposing ESEM and bifactor ESEM as alternative approaches to CFA in order to account for the cross-loadings as well as students' response styles in the factor structure. Indeed, although CFA indicated acceptable model fit statistics, ESEM and in particular bifactor ESEM were empirically preferred. Accounting for conceptual overlaps and imperfect item-factor links in the factor structure leads to more appropriate representations of students' perceptions than models assuming perfect item-factor links (e.g., Wagner et al. 2013). ESEM consequently provides a flexible approach to describe the structure of perceived instructional quality based on substantive theory (Marsh et al., 2014).

In addition to addressing the challenge of potential cross-loadings, the bifactor models were capable of disentangling the general and specific factors of students' perceptions. This strength may become particularly important for researchers who would like to correct students' responses on the instructional quality items from culturally driven response styles and biases (He and van de Vijver, 2013). Moreover, bifactor modeling allows substantive research on general and specific perceptions of teaching practices (Charalambous et al., 2014). Generally speaking, given that imperfect item-factor links and the generality-specificity distinction may represent substantive issues, our modeling framework (see Table 1) provides an agenda that can easily be used to evaluate the degree to which these issues affect findings on factor mean differences or relations to other constructs. Moreover, the investigation of the factor structure can be regarded as an essential part of evaluating the match between the theoretical assumptions on the structure of students' perceptions and the empirical evidence obtained from the data (McCoach et al., 2013).

Our second research question was concerned with the measurement invariance of the previously identified factor structure across three countries. We were able to show that the differentiation into the three factors of individual students' perceived instructional quality was generalizable across the three countries. This finding indicates the persistence of these factors even in international contexts (Klieme, 2013). Moreover, given that strict invariance was met, the measures of students' perceptions were fully comparable and can be used to evaluate the different levels of perceived instructional quality (Millsap, 2011). The fact that measurement invariance could be established may also partly be explained by the similarities in language and culture of the three selected countries, which may have led to the positive situation that students understand the items on instructional quality similarly and therefore reduce response bias (He and van de Vijver, 2013). Besides the comparability of the measurement across countries, our study also provided strong evidence on the robustness of the invariance findings across different modeling approaches. Consequently, there was no indication of methodological bias with respect to comparability (Duncan et al., 2014).

It should be briefly noted though, that, for all modeling approach, the AIC and the BIC, even though they are very rarely used for invariance testing, did not suggest that scalar invariance was the most preferred model (see Table 5). As most applied researchers rely on the well-established criteria for the CFI, TLI, RMSEA, and SRMR (Cheung and Rensvold, 2002; Chen, 2007), we used these criteria as well, but the inconsistency between the AIC and BIC values and these well-established criteria might be an interesting topic for further research on the characteristics of AIC and BIC for evaluating measurement invariance.

In addition to the confirmatory results on the factor structure, the correlations among the three factors confirm previous findings. More precisely, existing research suggested lower correlations between perceived teacher support and classroom management, classroom management, and cognitive activation than the correlations between teacher support and cognitive activation (Wagner et al., 2013; Fauth et al., 2014). Indeed, perceptions of cognitively activating learning environments often go along with perceptions of teacher support, because cognitive activation in classrooms often requires teachers' support (Jonassen, 2011). This also explains the considerably low loadings of the perceived classroom management items on the general factor. This general pattern of relations between the factors was found using all modeling approaches.

Taken together, the examination of the factor structure of perceived instructional quality confirmed our expectations on the distinction between three factors. Moreover, we extended the modeling approach of individual students' perceptions to exploratory structural equation and bifactor modeling, and showed that these approaches provide flexible construct representations.

### Relations among perceived instructional quality and educational outcomes

Addressing Research Question 3 on the relations among perceived instructional quality and educational outcomes, we identified patterns of relations that were also found in previous studies. More precisely, we observed that self-concept and motivation were significantly explained by the three factors of perceived instructional quality, whereas achievement was only explained by perceived classroom management in most of the countries (e.g., Kunter et al., 2007; Rakoczy et al., 2008; Fauth et al., 2014; Dietrich et al., 2015). Interestingly, these results were robust across countries, implying that they may persist in cultures that are similar to the ones examined in the present study. Nevertheless, the multi-group approaches applied to the student-level data provide opportunities to study variation in the relations across groups (e.g., Marsh et al., 2009). We argue that the bifactor models are particularly useful in describing the relations to external variables, because the resulting regression coefficients are not biased due to the multicollinearity of predictors.

Finally, we point out that investigating the relations between individual perceptions of instructional quality and individual, educational outcomes represents an approach that considers individual differences in these variables to be important. This perspective might be in contrast to what has been argued in the context of climate and contextual effects. Specifically, there has been the argument that perceptions of instruction are to be solely used as classroom- or some kind of group-level aggregates (e.g., Marsh et al., 2012). Although we completely agree with this position for research questions that are concerned with the characteristics of the learning environment (Lüdtke et al., 2009), emphasizing the need for thorough multilevel modeling approaches (e.g., Marsh et al., 2012; Scherer and Gustafsson, 2015), we believe that studying inter-individual differences in students' perceptions is still a reasonable endeavor. Part of the reasoning for our standpoint is that considerably strong relations to educational outcomes can be found which do have individual variation that is not considered to be mere error or disagreement. In fact, these relations at the student level are quite robust across a number of educational large-scale studies and countries (e.g., PIRLS, PISA, and TIMSS). Henceforth, we would like to stimulate a critical discussion about the meaning and importance of individual students' perceptions of instructional quality.

### Limitations and future directions

When interpreting the results of the present study, some limitations need to be considered. First, given that the PISA 2012 data do not contain any information on classroom clustering due to the sampling design, the closest approximation of aggregating students' perceptions would have been the school level (OECD, 2013b). This sampling design issue in PISA 2012 challenges the examination of educational effectiveness models (Klieme, 2013). Using school- rather than classroom-aggregates changes the interpretation of perceived instructional quality substantially; the school-aggregated perceptions provide a criterion of the instructional environment that is similarly perceived by all students within *the same school* but in *different classrooms* (Lazarides and Ittel, 2012). We therefore encourage large-scale educational assessments to gather some information on classroom clustering in future surveys. We stress that extending the modeling approaches that were used in the present study to multilevel structural equation models would allow us to address typical questions of teaching effectiveness (e.g., How do different aspects of instructional quality affect students' educational outcomes?).

Second, in the current study, we did not specifically test for differential item functioning in order to sort out which items may work differently across the three countries. In this context, we encourage systematic investigations of cross-country measurement bias in perceived instructional quality (for an introduction into the concept of “measurement bias,” please refer to Jak et al., 2014).

Third, our sample comprises three countries, and so our findings related to measurement invariance may only apply for these countries. We would therefore like to encourage further research on measurement invariance issues using international large-scale data to perform analysis on different clusters of countries (e.g., the Nordic cluster, the Eastern European cluster, or the Asian cluster, etc.). As educational systems, languages, cultures, and economic systems vary, the comparability of educational measures across these systems may be compromised (Bulle, 2011).

## Conclusion

The findings of our study provided strong support for our expectations on the factor structure, measurement invariance across countries, and the relations to external variables regarding individually perceived instructional quality. We found evidence for the distinction between three factors of students' perceptions which relate to perceived teacher support, cognitive activation, and classroom management; three of the most crucial elements of instructional quality. Given that this result was robust across the three selected countries (Australia, Canada, and the USA), we conclude that students are generally able to differentiate between the three factors, irrespective of potential biases that might be due to educational differences in our data. However, this differentiation is not perfect, as indicated by the preference for more complex latent variable models that account for potential overlaps between the factors at the student level. As a consequence, we further conclude that modern psychometric approaches such as exploratory structural equation and bifactor modeling may represent the nature of students' perceptions more appropriately than traditional approaches which assume perfect links between items and factors. Moreover, we point out that bifactor models are particularly useful for disentangling the general and specific components of perceived instructional quality. On the basis of our findings, we encourage researchers to consider abandoning unnecessarily strict assumptions on the factor structure.

From an educational perspective, the present study identified individual differences in students' perceptions across countries that provide valuable information on how instructional quality is perceived. These findings have important implications for future research on linking instructional quality to student achievement, as they form the basis for studying cross-country differences at the student, classroom, or school level.

## Author contributions

All authors listed, have made substantial, direct and intellectual contribution to the work, and approved it for publication.

### Conflict of interest statement

The authors declare that the research was conducted in the absence of any commercial or financial relationships that could be construed as a potential conflict of interest.

## Footnotes

^1^We note that despite our decision to treat students' responses continuously, we checked the factor structure and measurement invariance for robustness across the data treatment. Using the WLSMV approach, we could not identify large deviations from the findings obtained from the continuous MLR approach. Neither our conclusions nor the robustness of the results across the three selected countries seemed severely threatened.
