# Supplementary material for: Evaluating Individual Students' Perceptions of Instructional Quality: An Investigation of their Factor Structure, Measurement Invariance, and Relations to Educational Outcomes
Source: Front Psychol. 2016 Feb 8;7:110. doi: 10.3389/fpsyg.2016.00110 (PMC4745267; doi:10.3389/fpsyg.2016.00110)
Supplement: Supplementary file 1 [file DataSheet1.docx]

***Supplementary Material***

**Evaluating students’ perceptions of instructional quality across three countries: An investigation of their factor structure, measurement invariance, and relations to educational outcomes**

**Ronny Scherer*, Trude Nilsen, Malte Jansen**

*** Correspondence:** Ronny Scherer: ronny.scherer@cemo.uio.no

1. **Supplementary figures**

TITLE: Single-group bifactor ESEM for the total sample

PISA 2012 data from AUS, CAN, and the USA

DATA: FILE IS PISA2012INQUA.dat;

FORMAT IS FREE;

VARIABLE: NAMES ARE CNT SCHOOLID STUDID ST77Q01 ST77Q02 ST77Q04

ST77Q05 ST77Q06 ST80Q01 ST80Q04 ST80Q05 ST80Q06

ST80Q07 ST80Q08 ST80Q09 ST80Q10 ST80Q11 ST81Q01

ST81Q02 ST81Q03 ST81Q04 ST81Q05 FSTUWT;

! ST77: teacher support, ST80: cognitive activation, ST81: classroom

! management

USEVARIABLES ARE ST77Q01-ST81Q05;

MISSING ARE ALL(-99);

CLUSTER = SCHOOLID;

! Specify the School-ID as the clustering variable

WEIGHT = FSTUWT;

! Students’ final weights

ANALYSIS: TYPE = COMPLEX;

ESTIMATOR = MLR; ! Use robust maximum likelihood estimation

ROTATION = TARGET(orthogonal);

! Target rotation with approximate zero-cross-loadings

! orthogonal rotation method

MODEL: ! Measurement model with target rotation

! Specific factors of perceived instructional quality

F1 BY

ST77Q01-ST77Q06

ST80Q01-ST80Q11~0

ST81Q01-ST81Q05~0(*1);

F2 BY

ST77Q01-ST77Q06~0

ST80Q01-ST80Q11

ST81Q01-ST81Q05~0(*1);

F3 BY

ST77Q01-ST77Q06~0

ST80Q01-ST80Q11~0

ST81Q01-ST81Q05 (*1);

! General factor of perceived instructional quality

FG BY

ST77Q01-ST81Q05(*1);

! Model modifications: residual correlations

ST77Q02 WITH ST77Q04;

ST80Q05 WITH ST80Q06;

ST81Q04 WITH ST81Q05;

OUTPUT: stdyx;

Supplementary Figure S1. M*plus* sample code for the Bifactor ESEM (total sample).

TITLE: Multi-group ESEM invariance tests (up to scalar invariance)

PISA 2012 data from AUS, CAN, and the USA

DATA: FILE IS PISA2012INQUA.dat;

FORMAT IS FREE;

VARIABLE: NAMES ARE CNT SCHOOLID STUDID ST77Q01 ST77Q02 ST77Q04

ST77Q05 ST77Q06 ST80Q01 ST80Q04 ST80Q05 ST80Q06

ST80Q07 ST80Q08 ST80Q09 ST80Q10 ST80Q11 ST81Q01

ST81Q02 ST81Q03 ST81Q04 ST81Q05 FSTUWT;

! ST77: teacher support, ST80: cognitive activation, ST81: classroom

! management

USEVARIABLES ARE ST77Q01-ST81Q05;

MISSING ARE ALL(-99);

CLUSTER = SCHOOLID;

! Specify the School-ID as the clustering variable

WEIGHT = FSTUWT;

! Students’ final weights

GROUPING IS CNT (36=Australia 124=Canada 840=USA);

! Specify the grouping variable

ANALYSIS: TYPE = COMPLEX;

ESTIMATOR = MLR; ! Use robust maximum likelihood estimation

MODEL = CONFIGURAL METRIC SCALAR;

! Specify the levels of invariance to be tested

ROTATION = TARGET(oblique);

! Target rotation with approximate zero-cross-loadings

! oblique rotation method

MODEL: ! Measurement model with target rotation

F1 BY

ST77Q01-ST77Q06

ST80Q01-ST80Q11~0

ST81Q01-ST81Q05~0(*1);

F2 BY

ST77Q01-ST77Q06~0

ST80Q01-ST80Q11

ST81Q01-ST81Q05~0(*1);

F3 BY

ST77Q01-ST77Q06~0

ST80Q01-ST80Q11~0

ST81Q01-ST81Q05 (*1);

! Model modifications: residual correlations

ST77Q02 WITH ST77Q04;

ST80Q05 WITH ST80Q06;

ST81Q04 WITH ST81Q05;

OUTPUT: stdyx;

Supplementary Figure S2. M*plus* sample code for the multi-group ESEM invariance testing.

1. **Supplementary tables**

**Supplementary Table S1.** Items measuring students’ perceptions of instructional quality (OECD, 2013b).

| *Item Wordings* | *Label* |
| --- | --- |
| **Teacher Support** |  |
| *How often do these things happen in your mathematics lessons? (0 = ‘never or hardly ever’, 1 = ‘some lessons’, 2 = ‘most lessons’, 3 = ‘every lesson’)* |  |
| The teacher shows an interest in every student’s learning. | ST77Q01 |
| The teacher gives extra help when students need it. | ST77Q02 |
| The teacher helps students with their learning. | ST77Q03 |
| The teacher continues teaching until the students understand. | ST77Q04 |
| The teacher gives students an opportunity to express opinions. | ST77Q05 |
| **Cognitive Activation** |  |
| *Thinking about your mathematics teacher that taught your last mathematics class: How often does each of the following happen? (0 = ‘never or rarely’, 1 = ‘sometimes’, 2 = ‘often’, 3 = ‘always or almost always’)* |  |
| The teacher asks questions that make us reflect on the problem. | ST80Q01 |
| The teacher gives problems that require us to think for an extended time. | ST80Q04 |
| The teacher asks us to decide on our own procedures for solving complex problems. | ST80Q05 |
| The teacher presents problems for which there is no immediately obvious method of solution. | ST80Q06 |
| The teacher presents problems in different contexts so that students know whether they have understood the concepts. | ST80Q07 |
| The teacher helps us to learn from mistakes we have made. | ST80Q08 |
| The teacher asks us to explain how we have solved a problem. | ST80Q09 |
| The teacher presents problems that require students to apply what they have learned to new contexts. | ST80Q10 |
| The teacher gives problems that can be solved in several different ways. | ST80Q11 |
| **Classroom Management** |  |
| *How often do these things happen in your mathematics lessons? (0 = ‘never or rarely’, 1 = ‘sometimes’, 2 = ‘often’, 3 = ‘always or almost always’)* |  |
| Students don’t listen to what the teacher says. | ST81Q01 |
| There is noise and disorder. | ST81Q02 |
| The teacher has to wait a long time for students to quiet down. | ST81Q03 |
| Students cannot work well. | ST81Q04 |
| Students don’t start working for a long time after the lesson begins. | ST81Q05 |

**Supplementary Table S2.** Descriptive statistics of items measuring students’ perceptions of instructional quality.

|  | Australia | | Canada | | USA | | Total sample | |
| --- | --- | --- | --- | --- | --- | --- | --- | --- |
| *Item* | *M (SD)* | *Range* | *M (SD)* | *Range* | *M (SD)* | *Range* | *M (SD)* | *Range* |
| ST77Q01 | 2.04 (0.92) | 0–3 | 1.97 (0.92) | 0–3 | 1.99 (0.89) | 0–3 | 2.00 (0.91) | 0–3 |
| ST77Q02 | 2.32 (0.85) | 0–3 | 2.35 (0.83) | 0–3 | 2.24 (0.86) | 0–3 | 2.33 (0.84) | 0–3 |
| ST77Q04 | 2.42 (0.79) | 0–3 | 2.43 (0.79) | 0–3 | 2.38 (0.8) | 0–3 | 2.42 (0.79) | 0–3 |
| ST77Q05 | 2.13 (0.96) | 0–3 | 2.09 (0.97) | 0–3 | 1.99 (0.96) | 0–3 | 2.09 (0.97) | 0–3 |
| ST77Q06 | 2.01 (0.99) | 0–3 | 2.02 (0.98) | 0–3 | 1.83 (1.01) | 0–3 | 1.99 (0.99) | 0–3 |
| ST80Q01 | 1.75 (0.91) | 0–3 | 1.88 (0.88) | 0–3 | 1.92 (0.88) | 0–3 | 1.84 (0.90) | 0–3 |
| ST80Q04 | 1.77 (0.86) | 0–3 | 1.84 (0.84) | 0–3 | 1.94 (0.85) | 0–3 | 1.83 (0.85) | 0–3 |
| ST80Q05 | 1.29 (0.93) | 0–3 | 1.51 (0.96) | 0–3 | 1.46 (0.98) | 0–3 | 1.43 (0.96) | 0–3 |
| ST80Q06 | 1.58 (0.89) | 0–3 | 1.71 (0.89) | 0–3 | 1.65 (0.93) | 0–3 | 1.66 (0.90) | 0–3 |
| ST80Q07 | 1.81 (0.91) | 0–3 | 1.98 (0.87) | 0–3 | 1.91 (0.90) | 0–3 | 1.91 (0.89) | 0–3 |
| ST80Q08 | 1.95 (0.94) | 0–3 | 1.96 (0.94) | 0–3 | 2.05 (0.93) | 0–3 | 1.97 (0.94) | 0–3 |
| ST80Q09 | 1.94 (0.93) | 0–3 | 2.04 (0.91) | 0–3 | 2.16 (0.88) | 0–3 | 2.02 (0.92) | 0–3 |
| ST80Q10 | 1.94 (0.88) | 0–3 | 2.05 (0.86) | 0–3 | 2.10 (0.86) | 0–3 | 2.02 (0.87) | 0–3 |
| ST80Q11 | 1.73 (0.90) | 0–3 | 1.95 (0.87) | 0–3 | 1.94 (0.86) | 0–3 | 1.87 (0.88) | 0–3 |
| ST81Q01 | 1.64 (0.92) | 0–3 | 1.80 (0.88) | 0–3 | 1.73 (0.89) | 0–3 | 1.73 (0.90) | 0–3 |
| ST81Q02 | 1.54 (0.96) | 0–3 | 1.73 (0.94) | 0–3 | 1.83 (0.91) | 0–3 | 1.67 (0.95) | 0–3 |
| ST81Q03 | 1.82 (0.98) | 0–3 | 1.99 (0.94) | 0–3 | 2.01 (0.92) | 0–3 | 1.93 (0.96) | 0–3 |
| ST81Q04 | 2.02 (0.91) | 0–3 | 2.13 (0.88) | 0–3 | 2.18 (0.87) | 0–3 | 2.10 (0.89) | 0–3 |
| ST81Q05 | 1.94 (0.97) | 0–3 | 1.94 (0.94) | 0–3 | 2.08 (0.91) | 0–3 | 1.95 (0.95) | 0–3 |

**Supplementary Table S3.** Factor means of perceived instructional quality across countries.

|  | **CFA/ ESEM** | | | **Bifactor CFA/ ESEM** | | | |
| --- | --- | --- | --- | --- | --- | --- | --- |
| *Means* | *Teacher support* | *Cognitive activation* | *Classroom management* | *General factor* | *Teacher support* | *Cognitive activation* | *Classroom management* |
| *Reference: Australia* |  |  |  |  |  |  |  |
| Australia | .00/ .00 | .00/ .00 | .00/ .00 | .00/ .00 | .00/ .00 | .00/ .00 | .00/ .00 |
| Canada | –.07*/ –.09* | .17*/ .19* | .20*/ .19* | –.01/ .01 | –.10*/ –.15* | .31*/ .28* | .21*/ .21* |
| Unites States of America | –.12*/ –.13* | .25*/ .26* | .24*/ .24* | .17*/ .19* | –.34*/ –.40* | .19*/ .15* | .20*/ .18* |
| *Reference: Canada* |  |  |  |  |  |  |  |
| Australia | .07*/ .09* | –.16*/ –.17* | –.18*/ –.18* | .00/ .01 | .11*/ .08 | –.30*/ –.32* | –.19*/ –.21* |
| Canada | .00/ .00 | .00/ .00 | .00/ .00 | .00/ .00 | .00/ .00 | .00/ .00 | .00/ .00 |
| Unites States of America | –.04/ –.04 | .08*/ .08* | .04/ .05 | .16*/ .17* | –.25*/ –.25* | –.10*/ –.11* | –.01/ –.02 |

*Note.* Variances of factors are standardized to 1. * *p* < .01

On the basis of the strict invariance models, we evaluated the differences in factor means across countries. As shown in Table S3, the CFA and ESEM approaches identified significantly lower means in perceived teacher support for Canada and the USA, compared to Australia, but significantly higher means for perceived cognitive activation and classroom management. Putting the Canadian sample as a reference revealed that only the means of cognitive activation were significantly higher in the USA. For the bifactor models, the same tendencies of factor means were found in the specific factors. Students’ general perceptions of instructional quality indicated the highest means in the USA.

We notice that the correlated-traits models (CFA and ESEM) and the bifactor models (Bifactor CFA, Bifactor ESEM) did not show significantly different results in mean comparisons. Nevertheless, the impact of accounting for cross-loadings could be identified locally with respect to the absolute values of effects (Table S3).
